# Duration of third stage labour and postpartum blood loss: a secondary analysis of the WHO CHAMPION trial data

**DOI:** 10.1186/s12978-021-01284-8

**Published:** 2021-11-14

**Authors:** Sumangala B. Chikkamath, Geetanjali M. Katageri, Ashalata A. Mallapur, Sunil S. Vernekar, Manjunath S. Somannavar, Gilda Piaggio, Guillermo Carroli, José Ferreira de Carvalho, Fernando Althabe, G. Justus Hofmeyr, Mariana Widmer, Ahmet Metin Gulmezoglu, Shivaprasad S. Goudar

**Affiliations:** 1 Department of Obstetrics and Gynaecology, S Nijalingappa Medical College, Bagalkot, Karnataka India; 2 Women’s and Children’s Health Research Unit JN Medical College, KLE Academy of Higher Education and Research, Belagavi, Karnataka India; 3 Statistika Consultoria, Campinas, São Paulo Brazil; 4 Centro Rosarino de Estudios Perinatales (CREP), Rosario, Argentina; 5 Reproductive Health and Research, World Health Organization, Geneva, Switzerland; 6 The Effective Care Research Unit, University of Witwatersrand and Walter Sisulu University, East London, South Africa; 7 Maternal and Perinatal Health, World Health Organization, Geneva, Switzerland; 8 Concept Foundation, Geneva, Switzerland; 9 University of Botswana, Gaborone, Botswana

**Keywords:** Third stage of labour, Postpartum blood loss, Duration, Heat-stable carbetocin, Uterotonics, Oxytocin

## Abstract

**Background:**

Obstetric haemorrhage continues to be a leading cause of maternal mortality, contributing to more than a quarter of the 2,443,000 maternal deaths reported between 2003 and 2009. During this period, about 70% of the haemorrhagic deaths occurred postpartum. In addition to other identifiable risk factors for greater postpartum blood loss, the duration of the third stage of labour (TSL) seems to be important, as literature shows that a longer TSL can be associated with more blood loss. To better describe the association between the duration of TSL and postpartum blood loss in women receiving active management of third stage of labour (AMTSL), this secondary analysis of the WHO CHAMPION trial data has been conducted.

**Methods:**

This was a secondary analysis of the WHO CHAMPION trial conducted in twenty-three sites in ten countries. We studied the association between the TSL duration and blood loss in the sub cohort of women from the CHAMPION trial (all of whom received AMTSL), with TSL upto 60 min and no interventions for postpartum haemorrhage. We used a general linear model to fit blood loss as a function of TSL duration on the log scale, arm and center, using a normal distribution and the log link function. We showed this association separately for oxytocin and for Heat stable (HS) carbetocin.

**Results:**

For the 10,040 women analysed, blood loss rose steeply with third stage duration in the first 10 min, but more slowly after 10 min. This trend was observed for both Oxytocin and HS carbetocin and the difference in the trends for both drugs was not statistically significant (p-value = 0.2070).

**Conclusions:**

There was a positive association between postpartum blood loss and TSL duration with either uterotonic. Blood loss rose steeply with TSL duration until 10 min, and more slowly after 10 min.

*Study registration* The main trial was registered with Australian New Zealand Clinical Trials Registry ACTRN12614000870651 and Clinical Trial Registry of India CTRI/2016/05/006969

## Plain Language Summary

The duration of the third stage of labour (TSL) seems to be an important risk factor for greater postpartum blood loss, as literature shows that a longer TSL can be associated with more blood loss. Active management of third stage of labour (AMTSL), included in the WHO guidelines for prevention of postpartum haemorrhage (PPH), is effective in reducing both the amount of postpartum blood loss and the duration of the third stage. To better describe the association between duration of TSL and postpartum blood loss in women receiving AMTSL, we conducted this secondary analysis of WHO CHAMPION trial data.

To assess the association between the duration of third stage of labour and postpartum blood loss, a subcohort of the CHAMPION modified ITT population was selected by excluding women with missing blood loss or missing TSL duration or TSL duration more than 60 min and women with interventions. Thus, the subcohort consisted of 10,040 women.

In women with vaginal birth and not receiving interventions for treating atonic PPH or other sources of bleeding, and with TSL duration up to 60 min, there was a positive association between duration of the TSL and postpartum blood loss. The blood loss rose steeply with duration in women with TSL of 10 min or less, while in women with longer TSL duration the slope was less steep.

There was no evidence of a difference between oxytocin and HS carbetocin in the pattern of association of duration of the TSL and blood loss.

## Background

Obstetric haemorrhage continues to be a leading cause of maternal mortality, contributing to more than a quarter of the 2,443,000 maternal deaths reported between 2003 and 2009. During this period, about 70% of the haemorrhagic deaths occurred postpartum [1]. In addition to other identifiable risk factors for greater postpartum blood loss, the duration of the third stage of labour (TSL) seems to be important, as literature shows that a longer TSL can be associated with more blood loss [2].

The delivery of the placenta is usually a rapid event, but the TSL may exceed 30 min in about 3% of women [2], which is traditionally accepted as the cut off for a prolonged TSL. Combs and Laros observed that there was no difference in the risk of haemorrhage, blood transfusion or curettage with TSL lasting less than 30 min, but the risk of these interventions rose progressively with the TSL lasting more than 30 min [2]. Other authors have noted that the risk of PPH increases with increasing TSL and starts even before 30 min. Magann et al. found that the risk rises after a TSL of 18 min and the odds of having PPH are 6 times higher when the TSL is more than 30 min [3].

AMTSL, included in the WHO guidelines for prevention of postpartum haemorrhage (PPH), is effective in reducing both the amount of postpartum blood loss and the duration of the third stage [4].

To better describe the association between the duration of TSL and postpartum blood loss in women receiving AMTSL, this secondary analysis of the WHO CHAMPION trial data has been conducted. The WHO CHAMPION trial was a large study enrolling 29,645 women across 23 sites in 10 countries in a randomized, double-blind, noninferiority trial comparing intramuscular injections of HS carbetocin (at a dose of 100 μg) with oxytocin (at a dose of 10 IU) administered immediately after vaginal birth [5]. The primary outcomes of the WHO CHAMPION trial were the proportion of women who experienced > 500 ml blood loss or had use of additional uterotonics; and the proportion of women who experienced > 1000 ml blood loss. The secondary outcomes included the proportion of women requiring use of additional interventions to stop bleeding and those having anticipated side effects amongst several others. The trial showed that HS carbetocin was noninferior to oxytocin for the prevention of blood loss of at least 500 ml or the use of additional uterotonic agents and failed to show noninferiority for the outcome of blood loss of at least 1000 ml. This paper aims to characterize the duration of the third stage of labour with AMTSL and to examine the association between the duration of TSL and postpartum blood loss among women with vaginal delivery, without interventions and with duration of TSL up to 60 min.

## Methods

This is a secondary analysis of the data from the WHO CHAMPION trial. The primary aim of this analysis was to describe the association between the duration of TSL and postpartum blood loss among women with AMTSL with vaginal delivery, without interventions and with duration of TSL up to 60 min, particularly to see whether there is a critical duration above which blood loss increases. The secondary aim of the analysis was to define the association described above separately for women receiving Oxytocin and for women receiving HS carbetocin.

For the purpose of this analysis, the duration of the TSL was defined as the time interval from the birth of the baby until the delivery of the placenta.

**Fig. 1:**
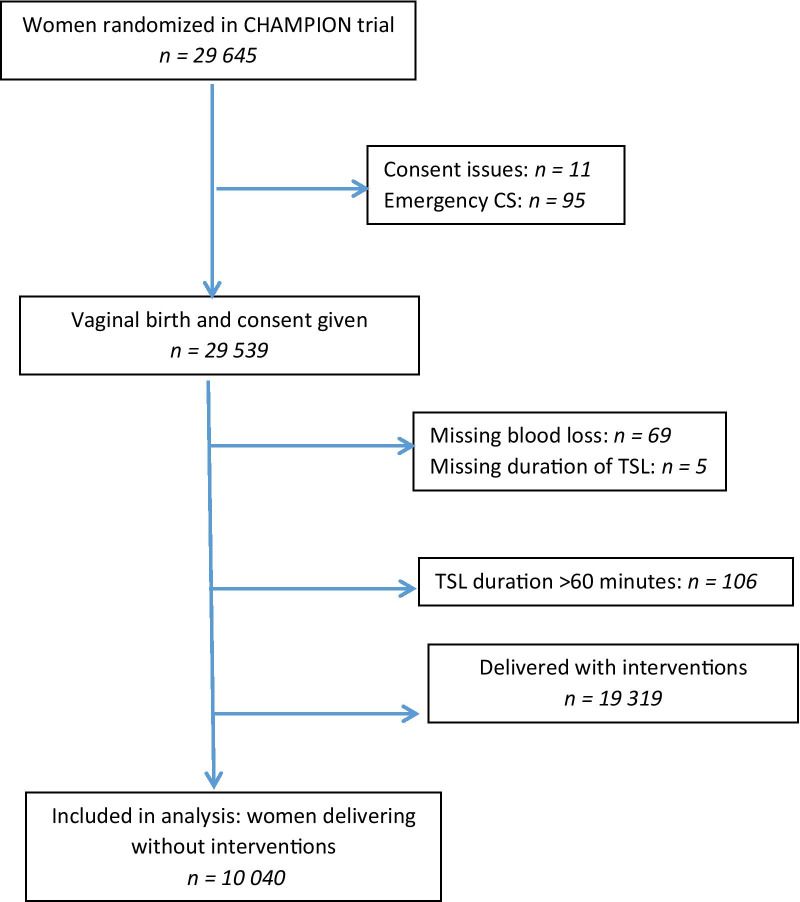
Study flowchart showing the selection of the sub cohort from the CHAMPION trial for the analysis of duration and blood loss

*Analysis Population* To assess the association between the duration of third stage of labour and postpartum blood loss, a subcohort of the CHAMPION modified ITT population (29,539 women with vaginal delivery and confirmed consent) was selected by excluding women with missing blood loss (69), with missing TSL duration (6) or TSL duration more than 60 min (106) and women with interventions (19,319) (Fig. 1). Thus, the subcohort consisted of 10,040 women.

The rationale for excluding women with interventions was as follows: TSL duration may be influenced by interventions to treat excessive bleeding. For example, excessive bleeding in the context of a retained placenta will induce treatment with uterotonics and probably a manual removal of placenta, which will determine the actual TSL duration according to the definition. To prevent this bias, the subcohort analyzed excluded women that had received any of the following interventions: episiotomy or perineal tear requiring suturing, use of additional uterotonics, manual removal of placenta, suturing cervical/ high vaginal tear, bimanual uterine compression, intrauterine balloon tamponade, exploration of the uterine cavity under anaesthesia, uterine or hypogastric artery ligation, uterine compression sutures, hysterectomy, manual/ surgical correction of uterine inversion, admission to Intensive Care Unit (ICU), or blood transfusion.

The rationale for excluding women with TSL duration > 60 min is explained under Statistical methods.

### Statistical analyses

The uterotonic received had been assigned at random in the CHAMPION trial within centers, to Oxytocin or HS carbetocin. As both the duration of TSL and the delivery with or without intervention were variables collected after the randomization took place, the relationship between blood loss and these factors was studied to explore the association and not to establish cause-effect relationships.

A generalized linear model was used to fit blood loss as a function of duration of TSL in minutes, center and arm, with the normal distribution and the log link function. The logarithmic transformation for blood loss was used based on the results from Carvalho et al. 2018 [6] showing that postpartum blood loss has a lognormal distribution. For TSL duration, the logarithmic transformation was used because the distribution of duration was very asymmetric. In a study of outliers including all data in the whole range of TSL duration, TSL duration values above 60 min (106 women, 0.36% of the CHAMPION modified intention-to-treat population) were found to have high leverage [7] and thus justified their exclusion from the analysis. As a first step, a model was fitted including the three two-factor interactions and the three-factor interaction, for the factors center, arm and TSL duration. Then, except for the interaction arm by TSL duration, only the interactions that were significant at the 5% level were kept in the final models. The interaction arm by TSL duration was kept in the model to obtain predicted values separately for the two drugs. Analysis of residuals was used to assess the quality of the fits.

Plots of the blood loss fitted values from the above models versus TSL duration were constructed, by arm, with 95% confidence bands obtained from the models by maximum likelihood. A test of interaction of TSL duration by arm was conducted.

## Results

**Fig. 2:**
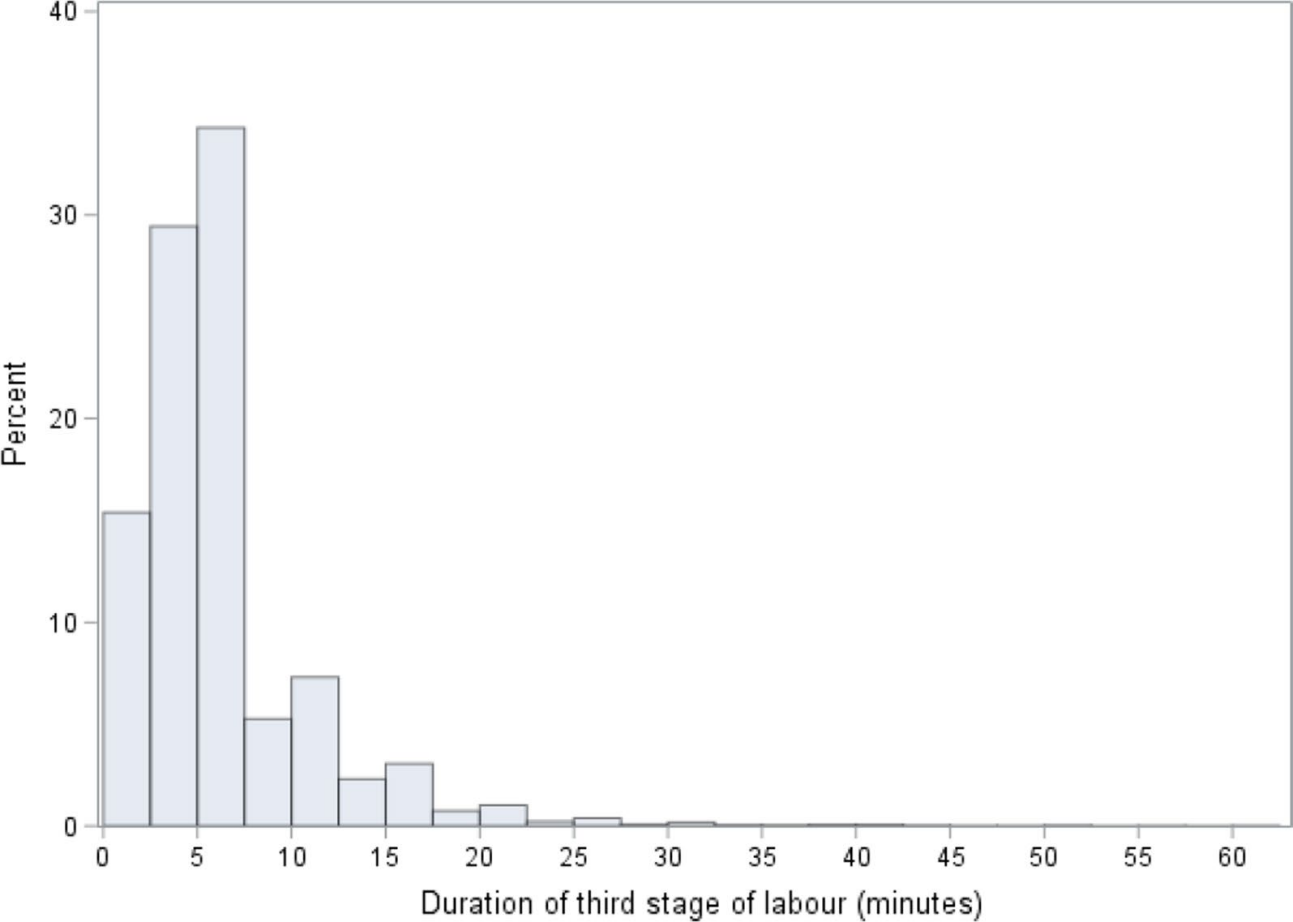
Distribution of the duration of the third stage of labour in minutes, from 0 to 60 min

Figure 2 shows the distribution of TSL duration within 60 min. The median duration of TSL was 5 min (interquartile range 3–7 min). Among the 10,040 women analyzed, there were 8927 (88.9%) women with duration within 10 min, 1074 (10.7%) with duration > 10–30 min and 39 (0.4%) with duration > 30–60 min.

**Fig. 3:**
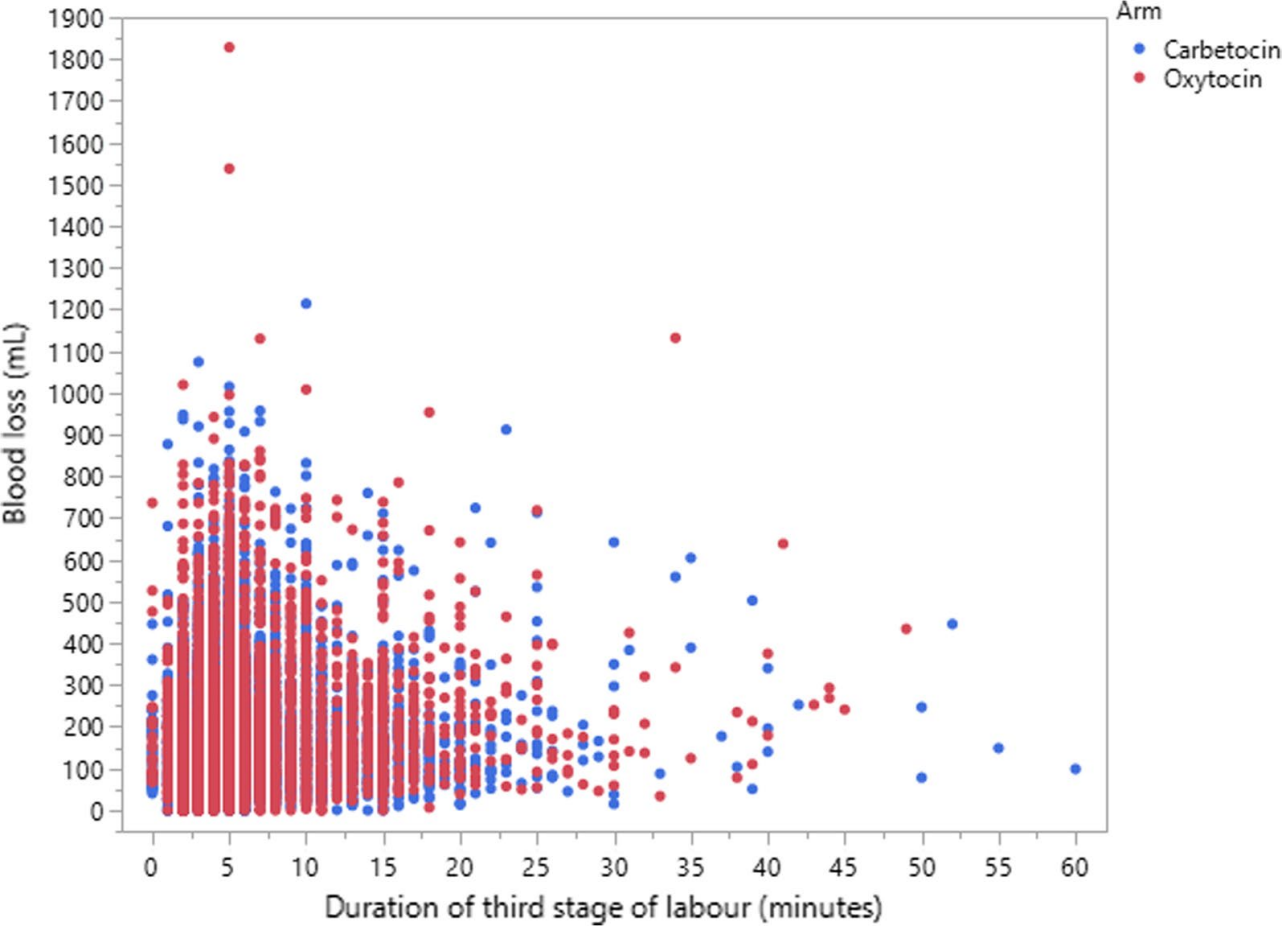
Scattergram of blood loss versus duration of the third stage of labour, by arm, in the range of duration 0–60 min for women delivering without interventions

To describe the pattern of blood loss with respect to TSL duration, a scattergram of blood loss versus duration, by arm, is presented in Fig. 3 for the range of 0–60 min.

**Fig. 4:**
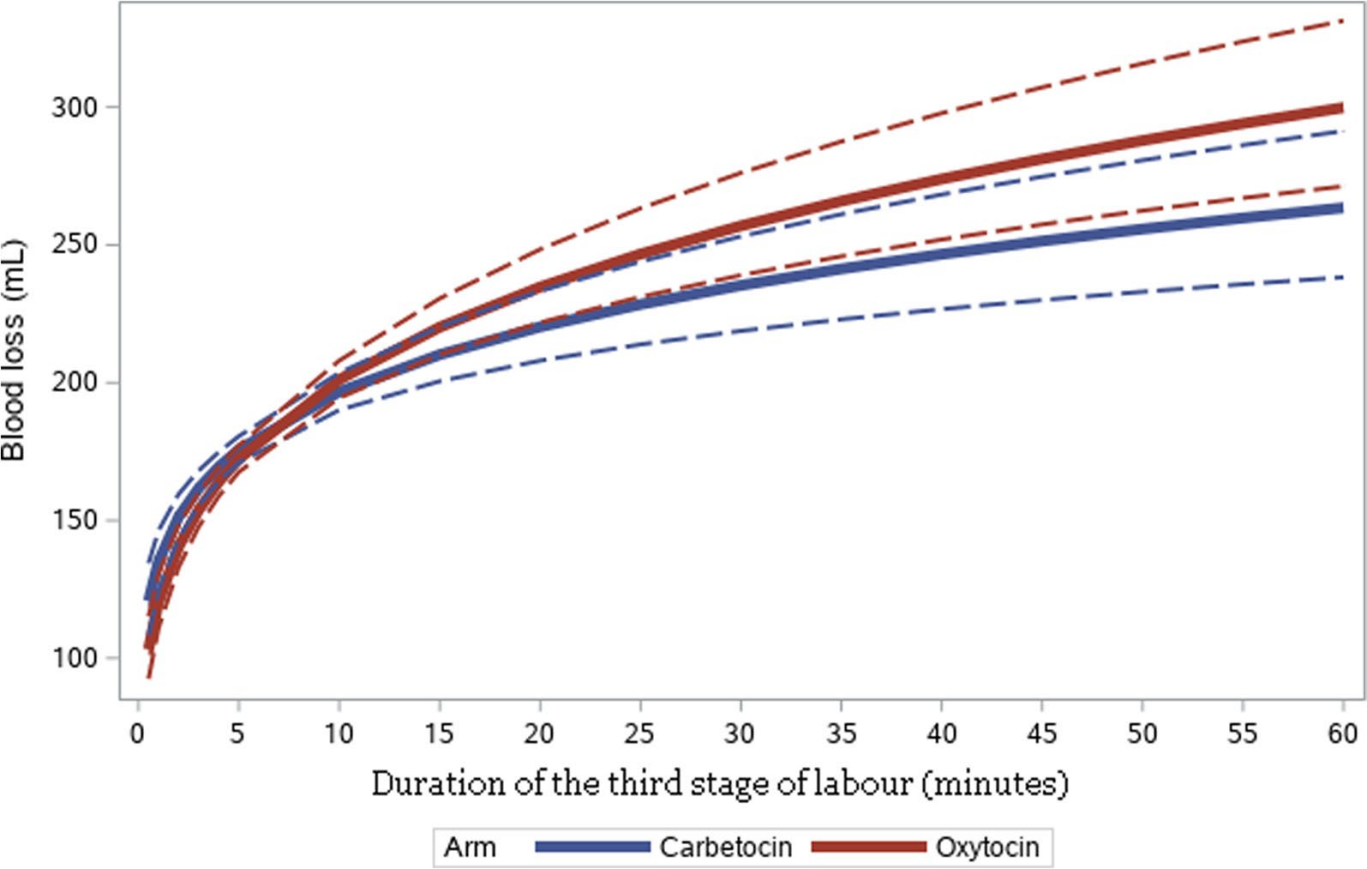
Predicted blood loss (median and 95% confidence limits) as a function of duration of TSL, by arm, for women delivering ‘without interventions’

The results of fitting a general linear model of the logarithm of blood loss on center, arm and logarithmic-transformed TSL duration is shown in Table 1. The predicted blood loss as a function of duration of TSL, for women delivering without interventions, by arm, is shown in Fig. 4. It can be seen that there is a positive association between blood loss and TSL duration in both arms (p-value = 0.0011 for the logarithm of duration). In the range of TSL duration within 10 min, corresponding to 88.9% of the women analyzed, the association between blood loss and TSL duration is very strong. After 10 min of TSL duration, the association curve is less steep. There is no suggestion of a critical duration above which blood loss increases, as the relationship seems smooth. The general pattern is similar for Oxytocin and Carbetocin, as shown by the overlapping confidence bands and the non-significance of the interaction of log duration by arm (p-value = 0.2070).

**Table 1:** Results of fitting a general linear model of the logarithm of blood loss on center, arm and logarithmic-transformed TSL duration

LR statistics for type 3 analysis			
Source	DF	Chi-square	Pr > ChiSq
Center	9	1432.55	< 0.0001
Arm	1	0.88	0.3470
Logduration	1	10.57	0.0011
Logduration*Arm	1	1.59	0.2070

## Discussion

*Main findings* Blood loss is affected by conditions causing increased bleeding, like episiotomy requiring suturing, genital trauma or surgical interventions [8]. We handled this source of variation by restricting the analysis in women having deliveries “with no interventions”.

There was a positive association between TSL duration and blood loss, among this group of women. The predicted value for blood loss increased steeply with TSL duration of at least 10 min (in which almost 90% of women are included), and more slowly after 10 min.

Regarding the association pattern of the two agents used in this study, differences might be expected due to the shorter half-life of oxytocin than HS carbetocin. Both Oxytocin and HS carbetocin seemed to have the same association curve up to 10 min, corresponding to almost 90% of the women. In women with a TSL of more than 10 min a slightly higher increase in blood loss for the same TSL duration was observed with Oxytocin than with HS carbetocin. However, the confidence bands overlap and the interaction of duration by arm is not significant, suggesting no evidence of a difference between the two drugs regarding association between duration of TSL and blood loss.

*Strengths and limitations* There are several strengths of this analysis. Our study included data of a large cohort of women delivering vaginally within the WHO CHAMPION trial, which is the largest PPH prevention study conducted to date. The multi-country, multi-center enrolments, the accurate postpartum blood loss measurement and high-quality data collection techniques employed, ensured a reliable database being available for this analysis.

However, there are also some limitations. We were not able to assess the association between blood loss and duration of the TSL beyond 60 min because of scarce data, as the proportion of women having a TSL lasting more than 60 min was very low (0.36%). Secondly, the models shown cannot be used for individual prediction: the 95% confidence bands shown in Fig. 4 are for predicted means. In the third place, since this was a post- hoc analysis, the study results should be interpreted with caution.

*Interpretation* Traditionally, TSL duration more than 30 min has been considered prolonged. Various authors have shown that the risk of PPH increases with an increasing duration of TSL and this happens at a much shorter duration than 30 min [9–11]. This was evident in our study too, as many women experienced very heavy blood loss with TSL of less than 10 min duration.

Our study explored the association between TSL duration and blood loss considering both variables as continuous, acknowledging the limitations to establish a causal relationship between TSL duration and more blood loss. Therefore, we did not compare the risk of PPH for different categories of TSL duration. We did not find a critical duration beyond which blood loss increases more steeply than before, on average. This is in contrast with results by other authors, where a longer duration of TSL was associated with a significantly higher risk of PPH. Studies have also described that a TSL longer than a particular duration is more likely to be associated with complications. According to Combs and Laros, with spontaneously delivered placentae, the incidence of PPH and blood transfusion is rather uniform in the first 30 min after delivery, after which it rises progressively [2]. Magann et al. found a significantly increased risk of PPH with a TSL beyond 18 min and Frolova et al. found a significant rise in PPH risk starting at TSL duration 20 min (15.9% at 20–24 min compared with 8.5% at less than 20 min) [3, 11]. In the current analysis, blood loss rose steeply with TSL duration up to 10 min, with the median blood loss in both arms at about 200 ml at TSL 10 min. With TSL duration between 10 and 60 min, the association curve was less steep, with median blood loss not exceeding 350 ml in either arm. Blood loss was not noted to be significantly heavier beyond any particular TSL duration and we did not identify any critical TSL duration threshold for increased blood loss.

Our findings of the association between blood loss and duration of the TSL by study drug should be interpreted in the context of the potential impact on clinical practice. The medications used in this study were stored under ideal temperatures, which is important for maintaining the potency of Oxytocin and which may not always be the case in actual clinical practice.

## Conclusion

In women with vaginal birth and not receiving interventions for treating atonic PPH or other sources of bleeding, and with TSL duration up to 60 min, there was a positive association between duration of the TSL and postpartum blood loss. The blood loss rose steeply with duration in women with TSL of 10 min or less, while in women with longer TSL duration the slope was less steep.

There was no evidence of a difference between Oxytocin and HS carbetocin in the pattern of association of duration of the TSL and blood loss.

The current study results should be interpreted with caution due to the fact that this was a post hoc analysis.

## Data Availability

The datasets generated during and/or analysed during the current study are available in the World Health Organization repository. The link to datasets can be requested to Mariana Widmer at widmerm@who.int.

## Abbreviations

PPH: Postpartum haemorrhage
HS: Heat stable
TSL: Third stage of labour
AMTSL: Active management of the third stage of labour
